# Book Reviews

**Published:** 1890-06

**Authors:** 


### Book Reviews.

### SPINAL CONCUSSION.

Surgically Considered as a Cause of Spinal Injury, and Neurologically Restricted to a certain Symptom Group, for which is suggested ERICHSEN'S DISEASE as one form of the Traumatic Neurosis.

BY S. V. CLEVENGER, M. D.

*Consulting Physician Reese and Alexian Hospitals, etc., etc., with Thirty Wood Engravings. F. A. Davis, Philadelphia. 1889.*

Twenty-five years ago Erichsen formulated a group of neurotic symptoms, and gave the name of Concussion of the Spine to the disease which they indicated. Since then, much discussion has taken place on the subject, and its importance, from both a medical and legal standpoint, has elicited a large amount of scientific *data* heretofore dormant. It would seem that the question has narrowed down to, not whether the pathological premises of Erichsen are correct or not, (though this point is still denied by some) but how to invariably determine the genuineness of those who claim to have received the injuries producing the disease.

The work before us deals most thoroughly in the subject, and is the best we have seen.

In the present state of our knowledge, there is nothing can be added to the book. It will go far toward making the road clear in legal proceedings, and to expose and prevent the impositions of fraudulent claimants.

The chapter on Diagnosis is very good, and treats largely of means and measures for exposing deception.

The author very appropriately suggests, in view of the fact that Erichsen was the pioneer in introducing, methodically, the disease to the consideration of the profession, that he should, as godfather at its christening, give it his name. As Erichsen's Disease, it would be much more appropriately and satisfactorily specified than by any other name.

The chapter on "Illustrative Cases of Spinal Disease," is very comprehensive and instructive, while the one on the recent discussions of Spinal Concussion present the subject in every aspect of scientific importance.

The work comes fully up to the demand, and the law and medical library, to be complete, cannot be without it.

### THE NEUROSES OF THE GENITO-URINARY SYSTEM IN THE MALE, WITH STERILITY AND IMPOTENCE.

BY DR. R. ULTZMAN.

Professor of Genito-Urinary Diseases in the University of Vienna. Translated by Gardner W. Allen, M. D., Surgeon in the Genito-Urinary Department Boston Dispensary. F. A. Davis, Philaphia and London. 1889.

Since Sayre advocated the connection between a certain group of neuroses and preputial deformities and adhesions, and recommended circumcision as a cure, there have been many medical Solons who have attempted to controvert these premises of experience by the query, "Why was it not the case before Sayre's day? Why did not Dupuytren, Nelaton, etc., find the same surgical necessity?" Of course, this is the stereotyped protest of the ultra skeptic, or the ignorant cynic, to whom all inventions and discoveries are diabolic innovations, and should not be allowed.

In the work before us, the pathology and treatment of neuroses of the genito-urinary system is fully entered into, and will be found very instructive. An unusual feature will be found in the absence of a table of contents and index, which takes from the work much of its attraction. It is a very valuable publication, however, and will do much toward elucidating and impressing the important details of this group of neurotic disturbances.

REVUE INTERNATIONALE DE BIBLIOGRAPHIE MEDICALE, PHARMACEUTIQUE ET VETERINAIRE, dirigie par le Docteur Jules Rouvier, Professeur de Clinique, &c., Beyrouth Lyrie, Vol. I, No. 1., April, 1890.

This volume is the first number of a periodical to be issued four times a year, which shall constitute an index to all works and articles pertaining to medicine, pharmacy and veterinary surgery, published in any part of the world. It occupies therefore the same place which is so ably filled in this country by the *Index Medicus* The value of such publications as this to all who write articles or works on medical subjects is too obvious to require comment. By no other means is it posible to investigate exhaustively the literature of any topic. Hence this *Revue* will doubtless be welcomed by many authors in the old world, to whom it will be more accessible than its American prototype. We wish it the success which it deserves.

